# Self-Evaluation in Childhood Social Anxiety Disorder: Effects of Repeated Exposure with Support Strategies

**DOI:** 10.1007/s10802-026-01422-5

**Published:** 2026-03-12

**Authors:** Nadine Vietmeier, Brunna Tuschen-Caffier, Julia Asbrand

**Affiliations:** 1 Department of Psychology, Humboldt-Universität zu Berlin, Berlin, Germany; 2 Department of Psychology, Albert Ludwig University of Freiburg, Freiburg, Germany; 3 Department of Psychology, Friedrich Schiller University Jena, Jena, Germany; 4 German Center for Mental Health (DZPG), Site Halle-Jena-Magdeburg, Jena, Germany; 5 University Clinic of Child and Adolescent Psychiatry and Psychotherapy, University Medical Centre EWL, Bielefeld University, Bielefeld, Germany

**Keywords:** Social phobia, Self-appraisal, Speech task, Cognitive bias, Parental support, Self-instruction

## Abstract

Children with social anxiety disorder (SAD) often perceive their social performance more negatively than external observers, reinforcing negative self-appraisals. Cognitive models suggest a vicious cycle between such distortions and heightened anxiety. This study tested whether repeated exposure to a social-evaluative task, paired with brief, ecologically valid support strategies, improves self-appraisal in children with SAD compared to baseline without support. Participants were 76 children (SAD: *n* = 33, *M*_*age*_ = 11.73, *SD* = 1.46, 64% female; healthy controls [HC]: *n* = 43, *M*_*age*_ = 11.40, *SD* = 1.45, 62% female), recruited from an urban setting in Germany. In two sessions one week apart, children delivered a 5-min speech to a peer video audience. In Session 1, they performed without support; in Session 2, they received either brief parental support or a self-instruction during preparation. After each speech, children rated their performance. Observer-rated performance was assessed by three independent, trained coders blind to group, session, and condition. Self-rated performance improved across sessions in both groups, but children with SAD consistently rated themselves more negatively than HC peers. Observer-rated performance did not differ between groups or across sessions. No differential effects emerged between support types. Negative self-appraisal is a core, though partially modifiable, feature of childhood SAD. Repeated exposure to social stress paired with brief support may modestly improve self-perception, though effects may reflect exposure rather than specific support. Targeting distorted self-appraisals remains a key clinical focus.

**Supplementary Information:**

The online version contains supplementary material available at 10.1007/s10802-026-01422-5.

Social anxiety disorder (SAD) is among the most prevalent anxiety disorders in childhood and adolescence (WHO World Mental Health Survey Collaborators et al., 2017). Characterized by an intense and persistent fear of negative evaluation in social or performance situations (American Psychiatric Association, 2013), SAD often leads to avoidance and distress. As a result, children with SAD may struggle with everyday social interactions, which can undermine their emotional, social, and academic development (Beesdo et al., 2009; Rao et al., 2007). SAD typically emerges in late childhood and often follows a chronic course when untreated (Salari et al., 2024), making it critical to understand how children with SAD perceive and navigate social situations to refine treatment and early intervention strategies. Peer-evaluative contexts—arguably the most socially relevant and emotionally salient setting for children and adolescents (Furman & Buhrmester, 1992)—have been examined in subclinical populations to understand their impact on self-related cognitions (e.g., Miers, 2021; Miers et al., 2009). Building on this work, the present study simulates such a context in a clinically diagnosed sample, using repeated social performance tasks in front of an age-matched video audience to examine changes in self-appraisal.

## Self-Rated Social Performance in Children with SAD

Cognitive models emphasize negative self-appraisal as a key factor in maintaining SAD. According to Clark and Wells (1995), individuals with SAD engage in heightened self-focused attention, constantly monitor for signs of failure, and interpret their own behaviors in a negative light. This ongoing cycle of negative self-evaluation reinforces anxiety and avoidance, preventing corrective experiences that could break the cycle and alleviate the disorder over time. While this dynamic has been well-documented in adults, there is growing evidence that similar patterns occur in children with SAD (e.g., Hodson et al.,^2008^; Leigh & Clark, 2018*)*.

However, findings on children’s self-evaluations have not been entirely consistent. Several studies have shown that children with SAD underestimate their social performance, perceiving themselves as less skilled or more awkward than their peers (Cartwright-Hatton et al., 2003; Krämer et al., 2011; Tuschen-Caffier et al., 2011). In contrast, Lau et al. (2023) found no significant group differences in self-rated performance between children with SAD and healthy controls (HC), based on data from a performance-based stress paradigm. This indicates that self-evaluative biases may vary depending on the nature of the social challenge—such as performing in front of an audience versus engaging in direct interaction—as well as on developmental stage. Such variability suggests that, although negative self-appraisals are a hallmark of SAD, their expression may fluctuate across contexts, potentially offering opportunities for modification through targeted interventions. At the same time, longitudinal work indicates that such self-evaluative tendencies can become increasingly stable over time (Miers et al., 2013, 2014), underlining the importance of studying self-appraisal processes early in development, before they consolidate into more enduring cognitive styles.

Building on these considerations, a key theoretical debate emerges: Are negative self-appraisals in children with SAD biased reflections of intact social skills, or do they reflect an accurate perception of actual social deficits? Cognitive frameworks emphasize the former, suggesting that anxiety interferes with access to existing abilities—resulting in a performance deficit (Clark & Wells, 1995). However, others argue that the self-appraisals may be accurate, reflecting genuine skill deficits (cf. Hopko et al., 2001). An integrated perspective assumes that both mechanisms may operate simultaneously—where cognitive bias and subtle social skill limitations reinforce each other over time (Lau et al., 2023; Spence & Rapee, 2016).

This distinction is not merely theoretical: if children with SAD objectively lack social competence, treatment must address skill acquisition—in addition to reducing anxiety and cognitive bias. While our study primarily builds on the performance-deficit perspective, the design also allows exploration of whether observable improvements occur across sessions—which could, in theory, also reflect learning effects consistent with a skill deficit (Hopko et al., 2001).If anxiety limits access to existing social skills, reducing it may help children with SAD perform more effectively and reevaluate their abilities more realistically. This assumption provides the foundation for brief support strategies aimed at improving performance-related self-appraisal.

### Brief Strategies to Reduce Anxiety and Improve Distorted Self-Appraisal

Cognitive models propose that modifying situational factors, such as increasing social support or self-efficacy, can break the cycle of negative appraisal and avoidance (Clark & Wells, 1995*)*. For example, parental support, as an external source of social buffering, has been shown to reduce stress in children (Taylor, 2012; Gottman & Katz, 1989). Similarly, self-instruction techniques foster internal coping by restructuring anxious thoughts (Schneider et al., 2022). Thus, brief strategies like parental support or self-instruction may offer scalable and accessible approaches to reduce anxiety and enhance self-appraisals (Clark & Wells, 1995).

In the present study, we focused on these two theoretically distinct yet complementary preparation strategies. Parental support may reduce children’s anxiety through processes such as emotional co-regulation and perceived social support (Hostinar et al., 2014), whereas self-instruction is expected to enhance autonomy, cognitive reappraisal, and self-efficacy (Bandura, 1997; Meichenbaum, 1977). In the present design, these strategies were used to probe the situational modifiability of self-evaluation rather than to compare their relative efficacy.

## Observer-Rated Social Performance in Children with SAD

Empirical evidence from independent observer ratings of social performance remains mixed. Some studies report no observable differences between children with SAD and healthy peers (e.g., Krämer et al., 2011), while others find poorer (e.g., Lau et al., 2023; Scharfstein & Beidel, 2015) or even better performance (Asbrand & Tuschen-Caffier, 2022) in children with SAD. Recent developmental work further underscores the importance of observable social behaviors for understanding social functioning in children with SAD. For instance, Klein et al. (2021) found that parent-rated social skills were markedly lower in children with SAD compared to those with other anxiety disorders, suggesting that subtle, parent-observed behavioral impairments may accompany self-evaluative biases. This evidence supports the notion that parent-observed and perceived aspects of social competence jointly contribute to children’s social adjustment and clinical outcomes.

Such inconsistency across studies may reflect methodological differences or the context-dependent nature of social performance impairments, particularly with regard to task characteristics and the type of audience involved (Asbrand & Tuschen-Caffier, 2022). Many studies have used standardized tasks without a direct audience present (e.g., Cartwright-Hatton et al., 2003), or with adult evaluators, as in the Trier Social Stress Test for Children (TSST-C; Buske-Kirschbaum et al., 1997) (e.g., Asbrand & Tuschen-Caffier, 2022; Krämer et al., 2011; Lau et al., 2023). However, children with SAD often struggle most in peer settings such as classrooms or school breaks (Ernst et al., 2023). Thus, designs lacking peer evaluation may overlook subtle impairments that are highly relevant in daily life (Miers, 2021; Miers et al., 2009). Ecologically valid paradigms that simulate real-life peer evaluation are essential to determine whether observed difficulties stem from genuine social skill deficits or are shaped by context.

## The Present Study

The present study examined both self- and observer-rated social performance in children with SAD and HC children across two sessions of a standardized speech task in front of a peer audience, allowing for the ecologically valid assessment of change over time. In session 1, children gave a speech without support. In session 2, they received either parental support or a self-instruction intervention prior to the speech task.

Our first aim was to examine whether children with SAD rate their performance more negatively than healthy peers, as reported in several previous studies (e.g., Cartwright-Hatton et al., 2003; Krämer et al., 2011; Tuschen-Caffier et al., 2011). Second, we hypothesized that repeated exposure to the task, accompanied by either parental support or self-instruction, would lead to improvements in self-rated performance, particularly in the HC group (Schneider et al., 2022; Taylor, 2012). In the SAD group, improvements were expected to be modest, reflecting persistent negative appraisal (Clark & Wells, 1995). Our third, exploratory aim was to examine group differences in observer-rated social performance at baseline and changes across sessions, given the mixed findings in the literature (Asbrand & Tuschen-Caffier, 2022; Klein et al., 2021; Krämer et al., 2011; Lau et al., 2023; Scharfstein & Beidel, 2015).

Clarifying the nature and malleability of negative self-appraisal in children with SAD may help refine cognitive models and support the development of early interventions targeting maladaptive self-evaluative processes.

## Methods

### Ethical Approvals and Consent Procedures

This study was approved by the Ethics Committee of the University of Freiburg, Germany (application no. 24/19), and conducted in accordance with the institutional guidelines and the ethical standards outlined in the 1964 Declaration of Helsinki and its later amendments. Informed consent was obtained from all participants and their legal guardians following detailed written and verbal explanations of the study procedures. Children in the clinical group were offered follow-up treatment at the department’s outpatient clinic or referrals.

### Study Design

This study was part of a larger cross-sectional research project comparing children with SAD and HC children on cognitive, behavioral, and physiological variables. The design and procedures are consistent with previously published reports from this project (Vietmeier et al., 2025). While physiological data were initially planned, technical issues precluded full inclusion. Prior to recruitment, eligibility criteria were preregistered with the German Clinical Trials Register (Clinical trial number: DRKS00018880, registration date: 05 July 2021) and remained unchanged. Most of a priori-defined outcomes have been reported in separate publications, including findings on cognitive variables (Vietmeier et al., 2025) and attentional biases (Vietmeier et al., under review). To ensure transparency, all publications include cross-references to related reports from the project. However, due to space constraints and specific research foci, we aimed for topic-specific manuscripts and can ascertain that detailed analyses on social performance—particularly the relationship between self- and observer-rated performance—have not yet been published.

The present manuscript reports secondary outcome analyses focused on self- and observer-rated social performance, preregistered prior to data analysis at the Open Science Framework (OSF; 10.17605/OSF.IO/QKS9Y).

Since the present study was part of a larger project, the target sample size (*N* = 92) was determined based on the primary research aims of that project. The self- and observer-rated social performance outcomes analyzed here were defined as secondary variables and analyzed after data collection was completed. To evaluate statistical power, we referred to a comparable study by Asbrand and Tuschen-Caffier (2022*)*, A power analysis using G*Power 3.1.9.7 (Faul et al., 2007) resulted in a required total sample size of *N* = 52 for a repeated measures ANOVA with 80% power (1 − β = 0.80) at α = 0.05 and *f* = 0.20. A power analysis for our exploratory analysis, with two between-subject factors and two within-subject factors, resulted in the same required sample size.

## Participants

Children aged 9 to 14 years were recruited via information letters based on population register data, local schools, social media, and newspapers. Inclusion criteria for the SAD group were a primary diagnosis of SAD according to DSM-5 criteria (DSM-5; American Psychiatric Association, 2013*)*. For the HC group, inclusion required the absence of any current or past mental disorder.

Exclusion criteria for all participants were a suspected or diagnosed autism spectrum disorder, acute suicidal ideation, intellectual disability (IQ < 80, approximated by school placement), use of medications likely to affect emotional or physiological responses (e.g., anxiolytics), and having a sibling already enrolled in the study. Diagnostic decisions were made based on structured clinical assessments and clinical judgement, under the supervision of licensed psychotherapists.

Of the original sample (see Vietmeier et al., 2025), 16 children were excluded from observer-based analyses of social performance due to technical issues leading to missing recordings (*n* = 15) or insufficient video quality (*n* = 1). This resulted in a final sample of 76 children (*n* = 33 SAD, *n* = 43 HC). All participants were fluent in German and lived in an urban German area. Comorbid diagnoses in the SAD group included: depressive disorders (*n* = 4), specific phobia (*n* = 4), insomnia (*n* = 1), agoraphobia (*n* = 1), and enuresis (*n* = 1). None of the children had received psychotherapy or pharmacological treatment specifically for SAD, although one participant was taking regular asthma medication. Participant characteristics are shown in Table 1.

**Table 1:** Participant characteristics

Characteristic	Group		Statistics
	SAD	HC	
Sample size (*n*)	42	46	
Mean age (*SD*), in years	12.30 (1.47)	11.88 (1.53)	*p* =.232^a^
Female (%)	69.7	48.8	*p* =.068^b^
Mean SDQ (*SD*)	15.36 (5.43)	8.86 (5.44)	*p* <.001^a^
Mean SDQ—mother (*SD*)	11.97 (5.00)	4.95 (4.10)	*p* <.001^a^
Mean SDQ—father (*SD*)	10.92 (5.31)	6.08 (4.66)	*p* <.001^a^
Mean SPAI-C (*SD*)	29.05 (9.40)	7.88 (6.84)	*p* <.001^a^
Mean SASC-R-D (*SD*)	57.45 (13.03)	34.83 (10.21)	*p* <.001^a^
Mean SASC-R-D—mother (*SD*)	59.97 (11.18)	32.38 (10.23)	*p* <.001^a^
Mean SASC-R-D—father (*SD*)	52.12 (10.58)	35.70 (10.93)	*p* <.001^a^
Mean DIKJ (*SD*)	21.76 (10.48)	8.79 (5.79)	*p* <.001^a^
School^1^			*p* =.181^b^
Elementary school (%)	30.3	37.2	
Integrated secondary school (%)	3.0	14.0	
Grammar school (%)	54.5	41.9	
Comprehensive school (%)	0.0	2.3	
Other (%)	12.1	2.3	
Not specified (%)	0.0	2.3	
Child lives primarily with…^1^			*p* =.427^b^
Both parents (%)	75.8	83.7	
Mother (%)	15.2	9.3	
Mother and male partner (%)	9.1	2.3	
Father and male partner (%)	0.0	2.3	
Not specified (%)	0.0	2.3	
Mother’s nationality^1, 2^			*p* =.087^b^
German (%)	84.8	95.3	
Other (%)	15.2	2.3	
Not reported (%)	0.0	2.3	
Father’s nationality^2, 3^			*p* =.706^b^
German (%)	72.7	79.1	
Other (%)	6.1	7.0	
Not reported (%)	21.2	14.0	
Average monthly net household income^1^			*p* =.164^b^
1,500-1,750€ (%)	0.0	2.3	
1,750-2,000€ (%)	3.0	0.0	
2,000–2,250€ (%)	3.0	0.0	
2,250-2,500€ (%)	0.0	7.0	
2,500-3,000€ (%)	12.1	11.6	
3,000–4,000€ (%)	30.3	9.3	
4,000–5,000€ (%)	15.2	18.6	
> 5,000€ (%)	36.4	48.8	
No response (%)	0.0	2.3	
Community size^1^			*p* =.429^b^
10,000–20,000 (%)	6.1	2.3	
100,000–200,000 (%)	3.0	0.0	
> 500,000 (%)	90.9	95.3	
Not reported (%)	0.0	2.3	

SDQ = Strength and Difficulties Questionnaire (Klasen et al., 2003), range: 0–40. SPAI-C = Social Phobia and Anxiety Inventory for Children (Melfsen et al., 2011), range: 0–42. SASC-R-D = Social Anxiety Scale for Children-Revised (Melfsen & Warnke, 2011), range: 18–90. DIKJ = Children’s Depression Inventory (Stiensmeier-Pelster et al., 2014), range: 0–56

^1^Based on maternal report (*n* = 76)

^2^Nationality data were only collected for parents; children’s nationality was not assessed

^3^Based on paternal report (*n* = 63). Some fathers did not complete the questionnaire, resulting in missing data. for certain variables

^a^Based on *t* test

^b^Based on *chi-square* test

## Stimuli and Measures

### Diagnostic Assessment

#### Clinical Interview

The *Diagnostic Interview for Mental Disorders in Children and Adolescents* (Kinder-DIPS; Schneider et al., 2017) is a structured diagnostic tool used to assess lifetime diagnoses, including both current and past conditions. It follows the criteria of the 10th revision of the International Statistical Classification of Diseases and Related Health Problems (ICD-10; World Health Organization, 2004) and the DSM-5 (American Psychiatric Association, 2013). The interview is conducted separately with both the child and a parent. All interviewers underwent extensive training to ensure the correct administration of the tool. Due to the Covid pandemic, the interviews were conducted online to reduce in-person contact and were recorded for supervision with a licensed psychotherapist. The Kinder-DIPS has demonstrated excellent interrater reliability for lifetime diagnoses (child report: κ = 0.90–0.98, parent report: κ = 0.88–0.98), high test-retest reliability (child report: 75–98%, parent report: 89–100% after one week), and has been validated with disorder-specific questionnaires.

#### General Psychopathology

The *Strength and Difficulties Questionnaire* (SDQ, Goodman, 1997) is a 25-item behavioral screening tool, divided into subscales for emotional symptoms, conduct problems, hyperactivity-inattention, peer relationships, and prosocial behavior. Each subscale consists of five items rated on a 3-point scale (‘not applicable’, ‘partially applicable’, ‘clearly applicable’). Both children and parents completed the German version (Klasen et al., 2003). While the overall scale's internal consistency was adequate, the subscales had lower reliability (α = 0.55-0.77; *r*_*tt*_= 0.58-0.67; Lohbeck et al., 2015). In the current sample, internal consistencies for the subscales were in line with the original publication (child report: α = .48-.82, mother report: α = .56-.86, father report: α = .41-.84).

#### Social Anxiety Symptoms

Social anxiety symptoms were assessed using the *Social Phobia and Anxiety Inventory for Children* (SPAI-C, Beidel et al., 2000) and the *Social Anxiety Scale for Children-Revised* (SASC-R-D, La Greca & Stone, 1993).

The SPAI-C (German version: Melfsen et al., 2011) evaluates behavioral traits associated with SAD. The self-report tool consists of 26 items, with nine sub-items that measure anxiety levels in different social situations (e.g., with familiar and unfamiliar peers, adults). Children respond on a 3-point Likert scale, ranging from ‘never or hardly ever’ to ‘almost always or always’. The SPAI-C has shown strong internal consistency and test-retest reliability in German samples (Cronbach’s α = 0.92; *r*_*tt*_ = 0.84). In the present study, internal consistency was excellent (α = 0.98).

Additionally, the SASC-R-D (German version: Melfsen & Warnke, 2011*)* was completed by children and parents. The 22-item scale includes two subscales: fear of negative evaluation (e.g., ‘I worry about what other kids think of me’) and social anxiety and distress (e.g., ‘I feel nervous when talking to kids I don’t know well’). Responses are given on a 5-point Likert scale, from ‘not at all’ to ‘all the time’. The German version has shown good test-retest reliability (*r*_*tt*_ = 0.67) and internal consistency (α = 0.67; Melfsen & Warnke, 2011). In this study, internal consistency was excellent for both child and parent reports (child report: α = 0.95, mother report: α = 0.97, father report: α = 0.94).

#### Depressive Symptoms

Depressive symptoms were measured using the *Children’s Depression Inventory* (CDI; Kovacs, 2003; German version: Stiensmeier-Pelster et al., 2014), a self-report tool where children rate the severity of 26 depressive symptoms on a 3-point Likert scale (from ‘not present’ to ‘strongly expressed’). Scores are strongly correlated with clinical evaluations and behavioral measures of depression. Internal consistency coefficients for the CDI have been reported as α = 0.87 (school sample) to α = 0.92 (clinical sample). In the current study, the internal consistency was excellent (α = 0.94).

### Laboratory Session Assessment

#### Self-Reported Anxiety

Children assessed their anxiety levels using a child-friendly *Visual Analogue Scale* (VAS; Schmitz et al., 2010, 2011). They marked a point on a 100 mm horizontal line anchored at ‘no anxiety’ and ‘extreme anxiety’; the distance from the left endpoint was measured (in mm) and transformed to a 0–10 scale for analysis.

#### Self-Rated Social Performance

Self-perceived social performance was assessed using the adapted version of the *Performance Questionnaire—Child* (PQ-C; Cartwright-Hatton et al., 2005), an 11-item scale measuring three domains: nervousness (e.g., ‘How nervous did you look?’), global impression (e.g., ‘How friendly did you look?’), and micro-behaviors relevant to social skills (e.g., ‘How loud and clear was your voice?’). Items are rated on a 4-point Likert scale from 0 (‘not very much’) to 3 (‘very much’). The German version of the adapted PQ-C was used (Krämer et al., 2011). Internal consistency for the micro-behaviors subscale was questionable (α = 0.70; ω = 0.64, 95% CI [0.52, 0.76]), while the nervousness subscale showed acceptable reliability (α = 0.73; ω = 0.74, 95% CI [0.65, 0.83]). The global impression subscale demonstrated excellent internal consistency, with α = 0.88 and ω = 0.88 (95% CI [0.83, 0.92]; see Supplementary Materials). Notably, similar limitations in internal consistency for the subscales have been reported in prior research (Asbrand & Tuschen-Caffier, 2022), underscoring the need for cautious interpretation.

#### Observer-Rated Social Performance

Objective social performance was assessed using the *Performance Questionnaire—Observer* (PQ-O; Cartwright-Hatton et al., 2005), a parallel version of the PQ-C completed by independent observers. The same German translation was used *(*Krämer et al., 2011), with nine items covering the same three domains. Higher scores reflect more positive evaluations of the child’s social performance. Internal consistency was acceptable for the micro-behaviors (α = 0.76; ω = 0.77, 95% CI [0.70, 0.85]) and global impression (α = 0.78; ω = 0.78, 95% CI [0.72, 0.86]) subscales. However, the nervousness subscale demonstrated poor internal consistency, with α = 0.20 and ω = 0.34 (95% CI [0.14, 0.55]; see Supplementary Materials). Nonetheless, all subscales were retained in the analysis to ensure comparability with prior research using the PQ-O. In line with our findings, previous studies (e.g., Asbrand & Tuschen-Caffier, 2022*)* have also reported modest to low internal consistencies for several PQ-O subscales, suggesting persistent psychometric challenges in observer-based assessment of social performance.

Observer ratings were provided by advanced graduate students in clinical psychology who were blind to group status, session, and condition. Raters received one day of training under supervision of the first and third author, including multiple training videos to ensure consistent use of the instrument. To control for potential order effects, video recordings were randomized for coding in a balanced sequence across group (SAD vs. HC), session (T1 vs. T2), and condition (parental support vs. self-instruction). Ratings were completed in five separate blocks, each consisting of 10–20 videos per coder, to allow for discussion and clarification of questions between the blocks without changing individual ratings. Each child was rated by all observers, and one session per child was rated twice by different coders to calculate interrater reliability. When more than one rating was available, final scores were averaged across coders (e.g., Asbrand & Tuschen-Caffier, 2022; Blöte et al., 2015a, b; Miers et al., 2009).

We examined interrater agreements using intraclass correlation coefficients (ICCs), applying a two-way random effects model with average measures (Koo & Li, 2016*)*. In Session 1, interrater reliability was good to excellent across all scales, with ICCs of 0.89 (95% CI [0.83, 0.93]) for the overall score, 0.92 (95% CI [0.87, 0.95]) for the micro-behaviors subscale, 0.76 (95% CI [0.65, 0.84]) for nervousness, and 0.75 (95% CI [0.55, 0.85]) for global impression. Reliability in Session 2 was slightly lower, with ICCs of 0.82 (95% CI [0.74, 0.88]) for the overall score, 0.81 (95% CI [0.72, 0.87]) for micro-behaviors, 0.64 for nervousness (95% CI [0.48, 0.76]), and 0.66 (95% CI [0.47, 0.79]) for global impression.

### Social Stress Task

A five-minute informal speech task adapted from Westenberg et al. (2009) was used to elicit social-evaluative stress. Children were given five minutes to prepare a speech on a self-chosen topic (e.g., hobbies, vacation, daily life). Topics were not restricted to ensure comparability in content familiarity and personal relevance across participants. Instructions emphasized that the speech should be ‘as interesting as possible so that the audience wants to get to know you better’. To maintain a social evaluative context, participants were informed that their presentation would be video recorded and watched live by peers, though a pre-recorded video of neutral-appearing age-matched children was used. Different audience recordings were shown in sessions 1 and 2 to increase ecological validity. If children hesitated for more than 20 s or struggled to begin with their speech, standardized encouragement was provided (Westenberg et al., 2009).

While the TSST-C (Buske-Kirschbaum et al., 1997) is often considered the gold standard for eliciting physiological stress responses, it typically involves formal adult evaluators. In contrast, peer-oriented tasks like the Leiden Public Speaking Task (Westenberg et al., 2009) offer higher ecological validity for studying social anxiety in youth, as peer evaluation represents a particularly salient context during childhood and adolescence. Our task was adapted accordingly to reflect real-world peer-evaluative situations.

### Experimental Conditions

In session 2, children were randomized (block randomization stratified by diagnostic group: SAD vs. HC) to parental support (SAD: *n* = 16, HC: *n* = 20) or self-instruction (SAD: *n* = 17, HC: *n* = 23). A chi-square test confirmed that the distribution of participants across support conditions did not differ between groups (χ^*2*^ (1, *N* = 76) = 0.03, *p* =.864), indicating successful stratified randomization.

In the parental support condition, the parent joined the child during speech preparation. The instruction was: ‘This time, your mother/father is allowed to help you prepare the speech’. No further guidance was provided to preserve natural interaction and maximize ecological validity. However, interactions were video-recorded and later reviewed to assess consistency across sessions. While content was not standardized, qualitative checks confirmed that most parents offered general encouragement (e.g., ‘You’ll do fine’, ‘Talk about your favorite hobby’) rather than specific scripting.

In the self-instruction condition, children used a personalized coping sentence created at the end of session (1) If needed, standardized prompts were given to support sentence generation (e.g., ‘What would you say to encourage a friend?’) and sentences were reviewed by the experimenter to ensure positive, relevant phrasing. The statement was written on a card (DIN A6) and placed on the table in front of the child during session (2) Children were instructed to repeat it (silently or aloud) during preparation.

### Procedure

Children completed two laboratory sessions exactly one week apart. Each session began with a 15-minute warm-up game. A 5-minute resting baseline (neutral landscape images; Asbrand et al., 2019) served as the pre-task reference period for subsequent measures (e.g., self-reported anxiety). It was followed by the anticipation phase: two minutes of silent preparation, then three minutes of preparation with audience exposure (via video, ‘that the audience can get to know you already’). This was followed by a 5-minute speech and a 5-minute recovery phase.

**Fig. 1:**
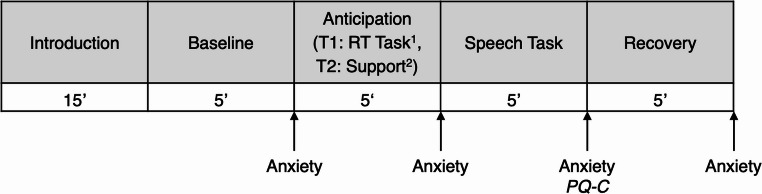
Procedure of laboratory sessions. *Note*. Anxiety = Children’s retrospect anxiety rating on a VAS scale (Schmitz et al., 2010, 2011). PQ-C = Performance Questionnaire—Child (Cartwright‐Hatton et al., 2003; Miers et al., 2009; German version: Krämer et al., 2011). T1 = Session 1. T2 = Session 2. ^1^The*RT Task* was not relevant to this study and will be reported elsewhere.^2^After group assignment, participants were randomized to a support condition during the anticipation phase (parental support or self-instruction).

At the end of session 1, all children generated personalized self-instruction sentences. Session 2 followed the same procedure as session 1 but included the support condition and a new speech topic. A reaction time task included in session 1 was not relevant to the present study. Upon completing the study, participants were debriefed and compensated with a 60€ voucher for children and a 40€ allowance for legal guardians. Figure 1 shows the procedure of both sessions.

### Statistics

All analyses were conducted using IBM SPSS Statistics for Windows (Version 29.0.2.0). Psychometric analyses, including internal consistency and reliability estimates, were performed in JASP (Version 0.19.3). The significance threshold was set at α = 0.05 (two-tailed), unless otherwise specified due to Bonferroni adjustment. Analytical procedures followed the preregistered plan. The primary dependent variables were self-rated and observer-rated social performance scores, assessed via the PQ-C and PQ-O subscales (nervousness, micro-behaviors, global impression). Items on the nervousness subscales were reverse-coded such that higher scores reflect better perceived or observed performance. Participants were excluded from individual analyses if more than 50% of the relevant questionnaire data were missing.

To test hypotheses on self-rated performance, a 2 (*group*: SAD vs. HC) × 2 (*session*: 1 vs. 2) × 2 (*condition*: parental support vs. self-instruction) mixed-design ANOVA was conducted for each PQ-C subscale. For observer-rated performance, the same design was applied, but only for the subset of participants with valid video data.

Post-hoc tests were Bonferroni-adjusted where applicable. Effect sizes are reported as partial eta-squared (η_*p*_^*2*^) and were interpreted according to conventional benchmarks (Cohen, 2013; Richardson, 2011), with η_*p*_^*2*^ values of 0.01, 0.06, and 0.14 representing small, medium, and large effects, respectively. For correlation coefficients, benchmarks proposed by Cohen (2013*)* were applied (*r* =.10 small, *r* =.30 medium, *r* =.50 large). Exploratory analyses included correlational analyses and interactions with additional covariates (e.g., age, comorbidities), which are explicitly labeled as such and not part of the preregistered core hypotheses.

## Results

To improve clarity, detailed results of internal consistency estimates, interrater reliability for observer ratings, baseline-adjusted ANCOVA analyses, regression models predicting change in self-rated performance, and correlations between reductions in self-reported anxiety during the speech phase and social performance are presented in the Appendix (see Supplementary Materials). The main text focuses on primary outcomes and key findings related to self- and observer-rated social performance.

### Manipulation Check

#### Self-reported Anxiety

**Fig. 2:**
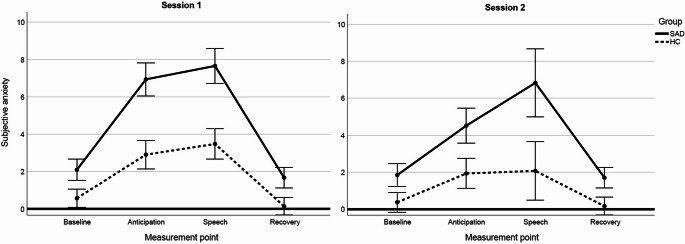
Progression of mean self-reported anxiety across task phases in children with SAD and HC children. *Note. *SAD= Social anxiety disorder group. HC = Healthy control group. Error bars: 95% CI. Self-reported anxiety was rated in retrospective on a VAS scale (range: 0-10 with higher values reflecting higher anxiety; Schmitz et al., 2010, 2011). Children with SAD reported significantly higher anxiety levels than HC across all phases (all *p*s< .001).

To confirm that the task elicited anxiety in the current sample (*n* = 76), we re-analyzed self-reported anxiety responses, which had previously been reported for the full study sample (see Vietmeier et al., 2025; see also Method section). A repeated-measures ANOVA was conducted with group (SAD vs. HC) as a between-subjects factor, and session (1 vs. 2), and time point (baseline, anticipation, speech task, recovery) as within-subject factors. The analysis revealed significant main effects of group and time point, as well as significant interactions between session and time point, and a three-way interaction between session, time point, and group (all *p*s < 0.001). Post hoc *t*-tests showed that children with SAD consistently reported higher levels of self-reported anxiety than HC at all time points (all *p*s < 0.001). Baseline anxiety levels did not differ significantly between sessions in either group (SAD: *t*(31) = 0.67, *p* =.508, *d* = 0.12, HC: *t*(42) = 1.29, *p* =.205, *d* = 0.20). In both groups and sessions, anxiety increased significantly from baseline to anticipation (SAD: *t*s ≥ −11.95, *p*s < 0.001, *d*s ≥ −2.08, HC: *t*s ≥ −6.87, *p*s < 0.001, *d*s ≥ −1.05), confirming that the task effectively elicited self-reported anxiety. Figure 2 illustrates the trajectory of self-reported anxiety across phases (baseline, anticipation, speech, recovery) for both groups and sessions.

#### Self-Rated Social Performance in Session 1

To assess whether children with SAD differed from HC in their self-perception of social performance before any support condition was applied, independent-samples *t*-tests were conducted on PQ-C scores from session 1. Children with SAD rated their social performance significantly more negatively than HC on the overall score as well as on all subscales (all *p*s ≤ 0.001; see Table 2).

**Table 2:** Baseline self-rated social performance between groups

PQ-C scale	Group	M	SD	95% CI (Lower)	95% CI (Upper)	t	df	*p*^a^	d
Overall score	SAD	3.04	1.31	1.04	2.27	5.35	74	< 0.001	1.24
	HC	4.70	1.36						
Micro-Behaviors	SAD	0.99	0.55	0.25	0.81	3.74	74	< 0.001	0.87
	HC	1.52	0.66						
Nervousness	SAD	1.38	0.72	0.36	0.95	4.39	74	< 0.001	1.02
	HC	2.04	0.58						
Global Impression	SAD	0.66	0.54	0.17	0.77	3.16	74	0.004	0.73
	HC	1.13	0.71						

Note. T1 = Session 1. SAD = Social anxiety disorder group. HC = Healthy control group. PQ-C = Performance Questionnaire—Child version *(* Cartwright-Hatton et al., 2003; Miers et al., 2009; *German version*: Krämer et al., 2011), self-reported social performance with overall score ranging from 0–9 and subscale scores ranging from 0–3

^a^Bonferroni-adjusted α = 0.005/4 = 0.0125

### Changes in Self-Rated Social Performance from Session 1 To Session 2

A repeated-measures ANOVA was first conducted on the overall self-rated social performance score, with *session* (1 vs. 2) as a within-subject factor and *group* (SAD vs. HC) as a between-subjects factor. Significant main effects were observed for both *session*, *F*(1,73) = 28.85, *p* <.001, η_*p*_^*2*^ = 0.28, and *group*, *F*(1,73) = 30.83, *p* <.001, η_*p*_^*2*^ = 0.30. However, the *session* x *group* interaction was not significant (*p* =.921). Post hoc *t*-tests revealed that both groups rated their overall performance significantly more positively in session 2 compared to session 1 (see Table 3).

**Table 3:** Differences in self-rated social performance across sessions

PQ-C scale	Group	M (SD)		95% CI (Lower)	95% CI (Upper)	t	df	*p*^a^	d
		T1	T2						
Overall score	SAD	3.05 (1.33)	3.71 (1.23)	−1.11	−0.22	−3.03	31	0.002	−0.54
	HC	4.70 (1.36)	5.33 (1.43)	−0.90	−0.37	−4.88	42	< 0.001	−0.74
Micro-Behaviors	SAD	0.98 (0.55)	1.16 (0.52)	−0.35	0.00	−2.00	31	0.027	−0.35
	HC	1.52 (0.66)	1.54 (0.62)	−0.16	0.12	−0.25	42	0.403	−0.04
Nervousness	SAD	1.41 (0.71)	1.70 (0.56)	−0.57	−0.03	−2.28	31	0.015	−0.40
	HC	2.04 (0.58)	2.34 (0.47)	−0.45	−0.15	−4.10	42	< 0.001	−0.63
Global Impression	SAD	0.66 (0.55)	0.85 (0.58)	−0.37	−0.02	−2.23	31	0.016	−0.40
	HC	1.13 (0.71)	1.45 (0.71)	−0.46	−0.18	−4.58	42	< 0.001	−0.70

Note. T1 = Session (1) T2 = Session (2) SAD = Social anxiety disorder group. HC = Healthy control group. PQ-C = Performance Questionnaire—Child version (Cartwright-Hatton et al., 2003; Miers et al., 2009; *German version*: Krämer et al., 2011), self-reported social performance with overall score ranging from 0–9 and subscale scores ranging from 0–3

^a^ Bonferroni-adjusted α = 0.005/4 = 0.0125

**Fig. 3:**
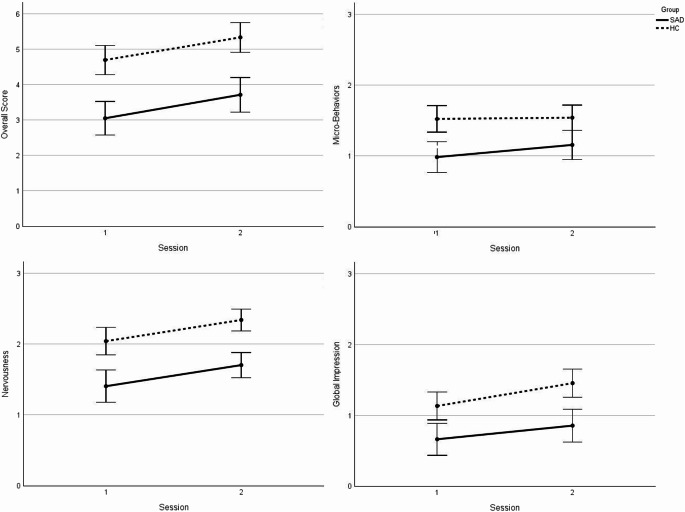
Changes in self-rated social performance from session 1 to session 2 by Groups

To further examine changes across specific dimensions, a two-way repeated-measures ANOVA was conducted, with *session* (1 vs. 2) and *scale* (micro-behaviors, nervousness, global impression) as within-subject factors, and *group* as a between-subjects factor. The assumption of sphericity was violated for the *session* x *scale* interaction (χ²(2) = 11.84, *p* =.003), so the Greenhouse-Geisser correction was applied (ε=0.90). Significant main effects were found for *session*, *F*(1,73) = 28.85, *p* <.001, η_*p*_^*2*^ = 0.28, *scale*, *F*(1,73) = 70.83, *p*<.001, η_*p*_^*2*^ = 0.49, and *group*, *F*(1,73)=30.83, *p* <.001, η_*p*_^*2*^ = 0.30. A significant *session* x *scale* interaction was observed, *F*(1.74, 126.78)=3.79, *p* =.031, η_*p*_^*2*^ = 0.05, but no other interactions, including the critical *session* x *scale* x *group*, reached statistical significance (all *p*s ≥ 0.198). Post-hoc analyses, following Bonferroni correction, revealed that only the subscales nervousness and global impression in the HC group were significant (see Table 3). Figure 3 illustrates the changes in self-rated social performance from session 1 to session 2 for both groups.

To control for initial between-group differences in self-rated social performance, and in line with preregistered analyses, ANCOVAs were conducted on change scores (session 2 minus session 1 self-rated social performance scores) using the respective session 1 outcomes as covariates. Group differences were not significant for the overall score or the micro-behaviors subscale (all *p*s ≥ 0.114). However, significant group differences were found for nervousness, *F*(1,72) = 11.83, *p* <.001, η_*p*_^*2*^ = 0.14, and global impression, *F*(1,72) = 5.03, *p* =.028, η_*p*_^*2*^ = 0.07, indicating distinct patterns of change in these subscales. For a detailed breakdown of these ANCOVA results, including regression findings, refer to the Appendix.

The self-rated social performance overall score at session 1 emerged as only significant predictor (β = − 0.30, *p* =.022) in relation to other potential predictors (e.g., age, SAD severity; Kunas et al., 2021) in a stepwise multiple linear regression (see Appendix). In Pearson correlations, reductions in self-reported anxiety during the speech phase from session 1 to session 2 were significantly correlated with improvements in both self- and observer-rated micro-behaviors for children with SAD, and with improvements in self-reported social performance (overall score and the nervousness subscale), as well as to improvements in observer-rated social performance (overall scale and the micro-behavior subscale) in HC (see Appendix).

### Explorative Analyses: Effects of Condition on Self-Rated Performance

To explore the potential effects of type of support condition on self-rated performance, we conducted a repeated measures ANOVA with *support condition* (parental support vs. self-instruction) and *group* (SAD vs. HC) as between-subjects factors, and *session* (1 vs. 2) as a within-subjects factor. To examine the subscales in more detail, a second repeated measures ANOVA was conducted, including *scale* (micro-behaviors, nervousness, global impression) as an additional within-subjects factor. Across both analyses, there was no main effect of *support condition* (both *p*s = 0.596) and no significant interactions involving *support condition* (all *p*s > 0.205). This suggests that the type of support did not significantly influence self-rated social performance across sessions.

### Explorative Analyses: Observer Ratings of Social Performance

#### Observer ratings in session 1

For group differences in observer ratings in session 1, two-tailed *t*-tests showed no significant group differences in the overall and the three subscales after Bonferroni adjustments (Bonferroni-adjusted α = 0.013; all *p*s ≥ 0.02).

#### Comparison between self- and observer ratings in session 1

Paired samples *t*-tests revealed that children with SAD rated their performance more negatively than the observers did across all scales, except for micro-behavior. In contrast, HC children rated only their global impression more negatively than observers did (see Table 4).

**Table 4:** Differences between self-rated and observer-rated performance in session 1

PQ scale	Group	M (SD)		95% CI (Lower)	95% CI (Upper)	t	df	*p*^a^	d
		Self	Observer						
Overall score	SAD	3.04 (1.31)	4.50 (1.42)	−1.95	−0.97	−6.11	32	< 0.001	−1.06
	HC	4.70 (1.36)	5.05 (1.32)	−0.80	0.09	−1.61	42	0.116	−0.25
Micro-Behaviors	SAD	0.99 (0.55)	1.10 (0.69)	−0.34	0.13	−0.90	32	0.187	−0.16
	HC	1.52 (0.66)	1.46 (0.63)	−0.17	0.30	0.56	42	0.579	0.09
Nervousness	SAD	1.39 (0.72)	1.88 (0.48)	−0.80	−0.19	−3.33	32	0.001	−0.58
	HC	2.04 (0.58)	1.99 (0.51)	−0.18	0.28	0.43	42	0.669	0.07
Global Impression	SAD	0.66 (0.54)	1.53 (0.61)	−1.06	−0.66	−8.82	32	< 0.001	−1.53
	HC	1.13 (0.71)	1.60 (0.56)	−0.70	−0.24	−4.05	42	< 0.001	−0.62

Note. T1 = Session (1) T2 = Session (2) SAD = Social anxiety disorder group. HC = Healthy control group. PQ = Performance Questionnaire (Cartwright-Hatton et al., 2003; Miers et al., 2009; *German version*: Krämer et al., 2011), either child version (self-reported; PQ-C) or other-version (observer-rated; PQ-O), with overall score ranging from 0–9 and subscale scores ranging from 0–3

^a^ One-sided *p*-values for children with SAD, two-sided for HC. Bonferroni-adjusted α = 0.005/4 = 0.0125

#### Change in observer ratings from session 1 to session 2

Repeated measures ANOVAs both on the observer-rated overall score and all subscales, with *group* (SAD vs. HC) as between-subjects factor and *session* (1 vs. 2) as within-subjects factor, revealed no significant effects for *session* nor any interaction with *session* (all *p*s ≥ 0.150), suggesting no observer-rated differences between session 1 and 2. Similarly, we found no significant effect for support conditions or any interaction with support condition (all *p*s ≥ 0.096), when adding support condition (parental support vs. self-instruction) as a second between-subjects factor.

## Discussion

The present study examined how children with SAD and HC perceive their own social performance during a structured speech task. The primary aim was to assess changes in self-rated performance across two sessions and to explore the potential impact of brief support strategies (parental support vs. self-instruction). Secondary exploratory analyses assessed observer ratings of social performance to provide an objective perspective on social behaviors, given the mixed findings in previous studies.

### Self-perception in Childhood SAD

Consistent with cognitive-behavioral models (Clark & Wells, 1995) and prior findings (e.g., Cartwright-Hatton et al., 2003; Krämer et al., 2011; Tuschen-Caffier et al., 2011), children with SAD consistently rated their own social performance lower than HC. This pattern was evident across all subscales of the PQ-C in session 1, and—extending previous studies—persisted even after a second exposure to the speech task.

Notably, both groups showed improvements in self-rated performance by session 2, suggesting that self-appraisals are at least partially malleable. However, group differences remained stable across sessions, with children with SAD continuing to rate themselves more negatively than their HC peers. While the overall effect of time was statistically significant in both groups, significant improvements on the subscale level were only found in the HC group (nervousness and global impression). In the SAD group, improvements on these subscales reached conventional significance levels (*p* <.05), but did not survive Bonferroni correction, suggesting that changes were less robust or more variable.

Nevertheless, effect sizes indicated meaningful change: for the overall self-rated performance score, improvements in the SAD group were small to moderate (*d* = 0.54), compared to moderate effects in the HC group (*d* = 0.74). ANCOVA results further confirmed that baseline differences in self-perception contributed to group differences, with HC showing more substantial improvements. Baseline self-perception also emerged as the most robust predictor of change, indicating a degree of temporal stability in self-views. This finding aligns with longitudinal studies indicating that initial self-appraisals in socially anxious youth tend to persist over time (Miers et al., 2013, 2014), potentially acting as cognitive anchors that limit spontaneous improvement.

While the support strategies used in this study were brief and not intended as full therapeutic interventions, they coincided with measurable improvements in self-appraisals across sessions. These findings reinforce the central role of distorted self-appraisals in childhood SAD and support their inclusion as key treatment targets in cognitive behavioral interventions (e.g., Hodson et al.,^2008^ Leigh & Clark, 2018).

At the same time, the absence of a no-support control group means that improvements may reflect repeated exposure rather than specific effects of the support conditions. For children with more entrenched negative self-views, additional targeted approaches—such as cognitive restructuring or video feedback—may be necessary to produce lasting changes. These have shown promise in modifying maladaptive beliefs socially anxious youth (cf. Morgan & Banerjee, 2006; Rapee et al., 2013).

### Brief Support Strategies: No Differential Effect

The absence of significant differences between the two support conditions (parental support and self-instruction) suggests that both strategies had similar effects on perceived social performance. This aligns with findings from our previous work with the same sample, which found comparable reductions in cognitive distortions (i.e., anticipatory rumination, self-focused attention, and post-event processing) under both conditions (Vietmeier et al., 2025).

These findings can be interpreted in light of the distinct theoretical mechanisms underlying each approach. Parental support may reduce situational anxiety through emotional co-regulation and perceived social buffering (Hostinar et al., 2014), whereas self-instruction is expected to promote autonomy, cognitive reappraisal, and self-efficacy (Bandura, 1997; Meichenbaum, 1977). The comparable outcomes observed here suggest that both externally guided and self-directed regulation can facilitate short-term reductions in anxiety and improve self-appraisal.

One interpretation is that support in general, regardless of its specific form, may be beneficial during socially demanding tasks. This would be encouraging for clinical practice, as it suggests that low-intensity strategies—whether externally provided (e.g., by parents) or internally applied (e.g., self-instruction)—can be flexibly integrated into interventions.

Moreover, the lack of differential effects might reflect heterogeneity in cognitive styles and social competence within both groups. Children may differ in the mechanisms they rely on—some profiting more from external reassurance, others from self-directed strategies. This highlights the need for more individualized approaches and aligns with a precision medicine perspective, moving beyond one-size-fits-all interventions. Thus, future studies should also consider contextual and cognitive factors that may shape the impact of brief support strategies. Although the present study focused on the situational effects of parental involvement and self-instruction, it would be informative to complement this paradigm with additional measures that capture the processes underlying each form of support. For instance, observational assessments of parent–child interactions could clarify how specific parental behaviors—such as reassurance, encouragement, or overcontrol—affect children’s immediate self-evaluative and emotional responses. Similarly, measures of children’s cognitive flexibility or self-efficacy could help elucidate individual differences in how self-instruction strategies are applied and internalized.

Nevertheless, these findings should be interpreted with caution. The small sample size, limited control over the content and implementation of support, and potential overlap between support types may have obscured true differences. In addition, the general improvement across both groups may reflect mechanisms such as increased task familiarity, reduced anticipatory anxiety, or expectation disconfirmation—consistent with exposure-based models of anxiety reduction (e.g., Pittig et al., 2023). Given these limitations and the absence of a no-support control condition, the present design was not intended or powered to directly compare the two strategies. Accordingly, the results should not be interpreted as evidence for equivalent efficacy of the two approaches, but rather as supporting the general modifiability of anxiety and self-evaluative processes across repeated exposures with support strategies. Including a no-support condition in future studies would allow for clearer conclusions about the specific effects of support.

#### Observer Ratings: Exploratory Insights

In line with earlier findings (Asbrand & Tuschen-Caffier, 2022; Krämer et al., 2011), observer ratings of social performance did not differ between groups and remained stable across sessions, despite consistent differences in self-appraisals. This contrast supports the performance deficit hypothesis: socially anxious children may know how to behave socially, but their anxiety inhibits performance under pressure (Clark & Wells, 1995; Hopko et al., 2001). This is consistent with findings from previous studies, which showed that internal experience and observable behavior often diverge under social stress (e.g., Asbrand & Tuschen-Caffier, 2022*)*.

However, measurement limitations must be considered. Some PQ-O subscales, particularly nervousness, showed low internal consistency, raising questions about the tool’s sensitivity. Although the PQ-O is widely used and offers pragmatic advantages, it may not capture subtle or socially salient behaviors. Alternative tools such as the Speech Performance Observation Scale for Youth (SPOSY; Blöte et al., 2015a, b)—a structured third-party observer rating instrument—may offer a more sensitive and ecologically valid alternative, as it captures aspects like self-disclosure and confidence. However, the SPOSY is considerably more comprehensive and time-intensive, which limits its feasibility in larger samples or studies with repeated assessments. In contrast, the PQ-O offers a more economical approach and ensures comparability with previous research, albeit at the cost of reduced sensitivity to subtle social behaviors. Additionally, studies using peer raters (e.g., Miers et al., 2010) have revealed that socially anxious children may appear more awkward or withdrawn to peers than adult observers detect using standard scales.

In our data, discrepancies between self- and observer ratings were particularly pronounced for interpretative aspects of performance (i.e., nervousness, global impression), while concrete behavioral indicators (i.e., micro-behaviors like voice clarity) were more aligned. This suggests that self-perception distortions may be strongest for ambiguous or internally focused cues, which are harder for observers to judge and more prone to interpretive bias—a well-documented cognitive feature of social anxiety (Heimberg et al., 2010).

The findings underscore the need for more ecologically valid, peer-sensitive and multimodal assessment tools that better capture socially meaningful performance deficits in children with SAD. Future research should include interactive, peer-based contexts (e.g., real-time peer feedback or classroom-based observations), and consider integrating behavioral coding and physiological measures to comprehensively assess the complex dynamics of social performance.

#### Clinical Implications

The current findings carry several implications for clinical practice. First, they underscore the importance of assessing and targeting social performance in peer-relevant contexts. Children with SAD may not show global social skill deficits, but their anxiety, avoidance behavior, and self-critical thoughts may inhibit the expression of otherwise intact abilities. Clinicians should be cautious in interpreting negative self- or parent-reports as evidence for skill deficits and consider observational data from structured, peer-salient settings (Blöte et al., 2015a, b; Miers et al., 2010).

Second, the modest yet consistent improvements in self-perception observed across repeated speech tasks suggest that brief, low-intensity strategies—such as parental support or self-instruction—may be feasible components of early intervention. These strategies may enhance children’s perceived competence and reduce anticipatory anxiety, especially when paired with structured exposure exercises *(*e.g*.*, Scaini et al., 2016). Still, their specific contribution remains unclear, as improvements may also reflect effects of task repetition or habituation.

Finally, given the limited responsiveness of negative self-appraisals in the SAD group and their predictive power for change, more targeted cognitive strategies may be required. Interventions such as video feedback, cognitive restructuring, or peer modelling should be considered to more directly challenge and modify negative self-beliefs (e.g., Hodson et al., 2008; Leigh & Clark, 2018).

#### Limitations, Strengths and Future Directions

Several limitations warrant consideration. First, the low internal consistency of some PQ-O subscales limits the precision of observer-based findings. While the PQ-O provides an economical and standardized tool, it may miss subtle yet socially relevant behaviors. Prior work has proposed alternative tools like the SPOSY (Blöte et al., 2015) or the use of peer raters (e.g., Miers et al.,^2010^) to address this. Nonetheless, our approach ensured feasibility and comparability across repeated sessions and with previous studies.

Second, the lack of a no-support control group limits causal interpretation. While comparing two active strategies allowed for a first controlled test, a third condition would have increased power demands substantially. Thus, the current design balanced practicality and scientific rigor.

Third, observer ratings were conducted exclusively by female graduate students. While this ensured rater consistency, it may have introduced gender-related perception biases. Future research should include mixed-gender or peer observers to better reflect real-world evaluation dynamics.

Fourth, as the sample was predominantly assigned female at birth, generalizability across sexes is limited. Given that girls typically show higher rates of social anxiety (Rapee et al., 2019) and tend to engage more in ruminative and verbal coping styles, whereas boys more often rely on avoidant strategies (Jose & Brown, 2008; Nolen-Hoeksema & Girgus, 1994; Rood et al., 2009), future research should examine whether such differences moderate responses to brief support strategies.

Fifth, the sample was recruited from an urban area in Germany and was predominantly characterized by German nationality and relatively high socioeconomic status (see Table 1). Thus, generalizability to more ethnically, culturally, and socioeconomically diverse populations may be limited; future studies should examine whether the observed patterns replicate in more diverse samples.

Finally, while the peer-audience paradigm increased ecological validity compared to camera-only or adult-based paradigms, it remained a simulation. The credibility of the peer evaluation was not systematically assessed, and reciprocal peer interaction was absent. Future studies should incorporate live peer-based settings to more accurately simulate real-world social evaluation (e.g., Miers et al., 2010).

## Conclusion

This study reinforces the central role of negative self-appraisal in childhood SAD, showing that children with SAD consistently rate their social performance more negatively than their healthy peers. These distorted self-evaluations, however, do not appear to correspond to observable performance deficits, suggesting that difficulties in SAD are driven by performance anxiety and negative self-perception rather than social skills deficits. Repeated exposure with brief support strategies led to modest improvements in self-appraisal, but further research is needed to clarify their distinct and long-term effects. The reliability issues with observer ratings highlight the need for alternative, more sensitive and potentially multimodal scales to assess social performance, possibly incorporating peer evaluations, to better capture the complex dynamics of SAD in children.

## Supplementary Information

Below is the link to the electronic supplementary material.

ESM 1 (DOCX 36.0 KB)

## Data Availability

Data cannot be shared publicly as this is not included in the informed consent by participants and the mental health data is particularly sensitive. However, deidentified participant data with annotations will be made available to other researchers upon reasonable request.
