# Supplementary material for: Self-Evaluation in Childhood Social Anxiety Disorder: Effects of Repeated Exposure with Support Strategies
Source: Res Child Adolesc Psychopathol. 2026 Mar 12;54(2):45. doi: 10.1007/s10802-026-01422-5 (PMC12982232; doi:10.1007/s10802-026-01422-5)
Supplement: Supplementary file 1 — (DOCX 36.0 KB) [file 10802_2026_1422_MOESM1_ESM.docx]

# **Appendix**

**Psychometric Considerations**

Internal consistency for the Performance Questionnaire (PQ; Cartwright‐Hatton et al., 2003; Miers et al., 2009; German version: Krämer et al., 2011), scales was evaluated using both Cronbach’s alpha and McDonald’s omega to account for potential violations of tau-equivalence and the use of short scales (Flora, 2020). For the PQ-C, internal consistency for the micro-behaviors subscale was questionable (α = .70; ω = .64, 95% CI [.52, .76]), while the nervousness subscale showed acceptable reliability (α = .73; ω = .74, 95% CI [.65, .83]). The global impression subscale demonstrated excellent internal consistency, with α = .88 and ω = .88 (95% CI [.83, .92]). For the PQ-O, reliability was acceptable for the micro-behaviors (α = .76; ω = .77, 95% CI [.70, .85]) and global impression (α = .78; ω = .78, 95% CI [.72, .86]) subscales. However, the nervousness subscale demonstrated poor internal consistency, with α = .20 and ω = .34 (95% CI [.14, .55]). Despite the partly poor to questionable reliability, we chose to analyze all the scales, including the nervousness subscale, to maintain comparability with previous research in this area.

We then examined interrater agreements using intraclass correlation coefficients (ICCs), applying a two-way random effects model with average measures (Koo & Li, 2016). In Session 1, interrater reliability was good to excellent across all scales, with ICCs of .89 (95% CI [.83, .93]) for the overall score, .92 (95% CI [.87, .95]) for the micro-behaviors subscale, .76 (95% CI [.65, .84]) for nervousness, and .75 (95% CI [.55, .85]) for global impression. Reliability in Session 2 was slightly lower, with ICCs of .82 (95% CI [.74, .88]) for the overall score, .81 (95% CI [.72, .87]) for micro-behaviors, .64 for nervousness (95% CI [.48, .76]), and .66 (95% CI [.47, .79]) for global impression. Taken together, these findings indicate that the overall and micro-behaviors scores of the PQ-O demonstrated solid interrater agreement and internal consistency, while the nervousness and global impression subscales require cautious interpretation due to lower reliability. The findings are discussed in the main text.

**Baseline-Adjusted Changes in Self-Rated Performance from Session 1 to Session 2**

To control for baseline differences between groups, ANCOVAs were conducted on change scores (session 2 - session 1) using baseline scores from session 1 as covariates. Group differences were not significant for the overall score, *F*(1,72) = 2.56, *p* = .114, η*_p_^2^* = .03, or the micro-behaviors subscale, *F*(1,72) = 0.09, *p* = .764, η*_p_^2^* = .00. However, significant group differences were found for nervousness, *F*(1,72) = 11.83, *p* < .001, η*_p_^2^* = .14, and global impression, *F*(1,72) = 5.03, *p* = .028, η*_p_^2^* = .07, suggesting distinct patterns of change in these subscales. Additionally, baseline scores were significant predictors of changes in self-perception across all subscales (overall score: *F*(1,72) = 10.48, *p* = .002, η*_p_^2^* = .13; micro-behaviors: *F*(1,72) = 18.64, *p* < .001, η*_p_^2^* = .21; nervousness: *F*(1,72) = 59.92, *p* < .001, η*_p_^2^* = .45; global impression: *F*(1,72) = 9.93, *p* = .002, η*_p_^2^* = .12), indicating that self-perceptions of social performance were moderately stable over time. The findings are discussed in the main text.

## **Explorative analyses: Predictors of Change in Self-Rated Social Performance**

To estimate the relative contribution of baseline scores and other potential predictors (Kunas et al., 2021) to changes in self-rated social performance, a stepwise multiple linear regression was conducted. The model included group (SAD vs. HC), age (in months), self-reported SASC-R-D score, mother-reported SASC-R-D score, father-reported SASC-R-D score, SPAI-C score, and baseline self-rated social performance as predictors, with the change score (session 2 – session 1) as the dependent variable. The overall model was significant, *F*(1,58) = 5.51, *p* = .022, with *R^2^* = .07, indicating that the predictors explained a modest amount of variance (7.1%) in the change score. Baseline performance emerged as the only significant predictor (β = -.30, *p* = .022) with lower initial self-ratings being associated with greater improvements in perceived social performance across sessions. None of the other predictors reached significance (all *p*s >.186). The findings are discussed in the main text.

## **Exploratory Analyses: Relationship Between Changes in Anxiety and Changes in Social Performance**

To assess the relationship between anxiety reduction (self-reported anxiety during speech) and changes in self- and observer social performance from session 1 to session 2, Pearson correlations were calculated for each group. For children with SAD, a reduction in anxiety from session 1 to session 2 was correlated significantly with improvements in both self- as well as observer-rated micro-behaviors (see Tab. A1). In contrast, for HC children, a decrease in anxiety was significantly associated with improvements in self-reported social performance (overall score and the nervousness subscale), as well as to improvements in observer-rated social performance (overall scale and the micro-behavior subscale; see Tab. A2).

To assess whether the relationship between changes in anxiety and changes in social performance differed between children with SAD and HC, we conducted a regression analysis for both self-rated and observer-rated overall performance scores. The interaction term (change in anxiety x group) was not statistically significant for either self-rated social performance, *B* = .13, *SE* = .03, *t* = 0.73, *p* = .471, or observer-rated social performance, *B* = .05, *SE* = .03, *t* = 1.68, *p* = .103. This suggests that the strength of the relationship between changes in anxiety and changes in social performance did not differ significantly between the two groups.

**Brief Discussion.** In both groups, anxiety increased in anticipation of the speech task, confirming the task’s effectiveness in eliciting subjective stress. However, reductions in anxiety from session 1 to session 2 showed only modest correlations with changes in social performance. For children with SAD, decreased anxiety was associated with observer-rated improvements in micro-behaviors, while in HC, anxiety reduction was linked to more generalized improvements in self- and observer-rated performance. Interestingly, the interaction between group and anxiety change was not statistically significant, suggesting that the relationship between anxiety and performance is more complex than initially hypothesized. Specifically, while anxiety and performance are related, they are not isomorphic—particularly in SAD, where distorted self-evaluation can persist despite reductions in anxiety. These findings align with Clark and Wells' (1995) model, which suggests that negative self-appraisal can maintain anxiety and prevent corrective experiences, even when anxiety levels decrease. Therefore, interventions focusing on reducing anxiety alone may not be sufficient to negative self-perception in children with SAD.

**Table A1**

*Correlation Coefficients Between Change Scores of Self-Reported Anxiety, Self- and Observer-Rated Performance in SAD Group*

| Variable | *M* | *SD* | 1 | 2 | 3 | 4 | 5 | 6 | 7 | 8 | 9 |
| --- | --- | --- | --- | --- | --- | --- | --- | --- | --- | --- | --- |
| 1. Anxiety During Speech | -0.83 | 7.12 | 1 |  |  |  |  |  |  |  |  |
| 2. PQ-C Overall Score | 0.66 | 1.24 | .13 | 1 |  |  |  |  |  |  |  |
| 3. PQ-C Micro-Behavior | 0.17 | 0.49 | .31* | .67** | 1 |  |  |  |  |  |  |
| 4. PQ-C Nervousness | 0.30 | 0.74 | -.09 | .72** | .06 | 1 |  |  |  |  |  |
| 5. PQ-C Global Impression | 0.19 | 0.49 | .16 | .78** | .62** | .25 | 1 |  |  |  |  |
| 6. PQ-O Overall Score | -0.01 | 1.10 | .29 | .29 | .12 | .23 | .28 | 1 |  |  |  |
| 7. PQ-O Micro-Behavior | -0.09 | 0.48 | .32* | .28 | .19 | .19 | .25 | .73** | 1 |  |  |
| 8. PQ-O Nervousness | 0.13 | 0.59 | .14 | .03 | .16 | -.13 | .13 | .62** | .13 | 1 |  |
| 9. PQ-O Global Impression | -0.05 | 0.53 | .17 | .32* | -.10 | .46** | .21 | .73** | .49** | .07 | 1 |

*Note.* SAD = Social anxiety disorder group (*n* = 32). PQ-C = Performance Questionnaire—Child version (Cartwright‐Hatton et al., 2003; Miers et al., 2009; German version: Krämer et al., 2011), self-reported social performance*.* PQ-O = Performance Questionnaire—Other version (Cartwright‐Hatton et al., 2003; Miers et al., 2009; German version: Krämer et al., 2011), observer-rated social performance. Change scores were calculated by subtracting session 1 from session 2 scores. Reported *p*-values are one-tailed.

**p* < .05, ***p* < .001.

**Table A2**

*Correlation Coefficients Between Change Scores of Self-Reported Anxiety, Self- and Observer-Rated Performance in HC Group*

| Variable | *M* | *SD* | 1 | 2 | 3 | 4 | 5 | 6 | 7 | 8 | 9 |
| --- | --- | --- | --- | --- | --- | --- | --- | --- | --- | --- | --- |
| 1. Anxiety During Speech | -1.41 | 2.09 | 1 |  |  |  |  |  |  |  |  |
| 2. PQ-C Overall Score | 0.64 | 0.86 | -.42** | 1 |  |  |  |  |  |  |  |
| 3. PQ-C Micro-Behavior | 0.02 | 0.46 | -.04 | .44** | 1 |  |  |  |  |  |  |
| 4. PQ-C Nervousness | 0.30 | 0.48 | -.49** | .65** | -.19 | 1 |  |  |  |  |  |
| 5. PQ-C Global Impression | 0.32 | 0.46 | -.24 | .75** | .01 | .37** | 1 |  |  |  |  |
| 6. PQ-O Overall Score | 0.19 | 1.37 | -.27* | .32* | .07 | .29* | .21 | 1 |  |  |  |
| 7. PQ-O Micro-Behavior | -0.02 | 0.57 | -.35* | .30* | .02 | .30* | .22 | .71** | 1 |  |  |
| 8. PQ-O Nervousness | 0.04 | 0.64 | -.08 | .13 | .08 | .21 | -.04 | .73** | .18 | 1 |  |
| 9. PQ-O Global Impression | 0.17 | 0.59 | -.20 | .30* | .06 | .17 | .32* | .84** | .48** | .44** | 1 |

*Note.* HC = Healthy control group (*n* = 43). PQ-C = Performance Questionnaire—Child version (Cartwright‐Hatton et al., 2003; Miers et al., 2009; German version: Krämer et al., 2011), self-reported social performance*.* PQ-O = Performance Questionnaire—Other version (Cartwright‐Hatton et al., 2003; Miers et al., 2009; German version: Krämer et al., 2011), observer-rated social performance. Change scores were calculated by subtracting session 1 from session 2 scores. Reported *p*-values are one-tailed.**p* < .05, ***p* < .001
